# Access to Food Establishments via Meal Delivery Applications: A Study of University and Non-University Settings in a Brazilian Metropolis

**DOI:** 10.3390/ijerph22091448

**Published:** 2025-09-18

**Authors:** Paloma Aparecida Anastacio Barros, Daniela Silva Canella, Paula Martins Horta

**Affiliations:** 1 Department of Nutrition, Nursing School, Federal University of Minas Gerais, Belo Horizonte 30130-100, Brazil; 2 Department of Applied Nutrition, Institute of Nutrition, Rio de Janeiro State University, Rio de Janeiro 20550-013, Brazil

**Keywords:** food access, meal delivery apps, food environment, organizational environment

## Abstract

This study aimed to characterize access to food establishments through a meal delivery application in university and non-university settings within a Brazilian metropolis. This cross-sectional study used data from a leading meal delivery platform. All establishments delivering to four public and four private university campuses in Belo Horizonte, Brazil were identified. For comparison, one corresponding non-university location was selected for each campus (yielding eight campus–location pairs). Each location corresponds to a central address within the paired neighborhood that was entered into the app to simulate delivery availability. Pairing criteria were based on geographic region and income levels. Information on establishment categories (classified by keywords), delivery distances, delivery fees, and geographic centrality was collected and analyzed descriptively. The number of available establishments ranged from 7176 to 11,440 across the assessed locations. Most establishments were categorized under keywords referring to snacks (e.g., burgers, savory snacks, pizza), regardless of location. Delivery distances ranged from 0 to 19.6 km, with shorter distances observed for university addresses and corresponding locations situated in central neighborhoods of the city, and longer distances for peripheral areas. Only 4.7% of establishments offered free delivery, and higher delivery fees were more frequent in non-university locations. No significant differences were observed between public and private universities. Food establishments are widely accessible via the app; however, central areas tend to have broader service coverage. Regardless of whether the location is a university or non-university setting, or whether it is central or peripheral, there is a predominance of establishments classified under keywords associated with unhealthy food options.

## 1. Introduction

The digital food environment is defined as the set of services and information related to food and nutrition available online, which can influence individuals’ dietary choices and eating behaviors [1,2]. Meal delivery applications (referred to as “apps”) constitute one of the key components of this environment and can be defined as platforms that manage the service, uniting, in the same system, the establishment that prepares the meal, the consumer who purchases it, and the delivery person who transports it to the end consumer [1,2,3]. Apps have played a significant role in the shift from home-cooked, traditional meals based on unprocessed or minimally processed foods to meals of lower nutritional quality [2,4].

These apps provide time savings in meal planning and preparation, as well as convenience and ease in obtaining ready-to-eat meals [4,5,6]. Moreover, the use of these apps increases the availability and accessibility of foods and beverages by expanding the geographic reach of deliveries, allowing consumers to order meals from establishments located beyond their immediate neighborhoods [2,7,8].

The menus offered through these apps may include traditional meals prepared using culinary techniques, as well as healthy options such as fruits and vegetables [2]. However, the predominant offerings are fast-food items and ultra-processed products, including sugar-sweetened beverages [2,3,4,5,8,9,10]. Consequently, the use of these platforms has been associated with adverse health outcomes, such as sedentary behavior, excess body weight, and an increased risk of chronic diseases [5].

A potential setting for the use of apps is organizational environments, such as universities [11]. In these settings, students and staff spend long hours and often have their meals on-site [11]. In Brazil, organizational food environments and their surroundings generally do not promote healthy eating, with a greater availability of ultra-processed foods compared to unprocessed or minimally processed foods and home-cooked meals [12,13]. The exposure to ultra-processed foods in Brazilian universities is increasing [14]. For instance, an audit of 54 food establishments at a public Brazilian university found a high prevalence of sugar-sweetened beverages (98%) and sweets and snacks (76%) [15]. In addition, persuasive advertising for these same products is widespread [12,15]. In workplace settings, the low availability of healthy foods has been identified as a factor associated with greater reliance on apps [9].

Given their capacity to attract large groups of people, university environments are of interest to both food establishments and app-based delivery services. Nevertheless, research on access to and use of these apps in Brazilian universities remains scarce, despite the country having more than 63 million users of such services [16]. Evidence from other countries highlights the relevance of app usage among university students [17,18,19]. For example, among students from 20 universities in Malaysia, 14.1% reported using these services once or twice per week [17], while 29% of students in Vietnam reported frequent app usage [18]. In China, low-nutritional-quality foods dominated delivery app offerings to university addresses and were the most popular among 20,000 students aged 18 to 30 years [19].

The aim of the present study is to characterize access to food establishments via meal delivery apps in both university and non-university environments within a Brazilian metropolis.

## 2. Materials and Methods

### 2.1. Study Design and Context

This cross-sectional observational study was conducted in the context of food establishments registered on Brazil’s leading meal delivery application. This company serves addresses at both public and private universities, as well as non-university locations, in Belo Horizonte, Brazil.

Founded in 2011, Ifood has shown significant growth nationwide [20]. In 2024, it held over 80% of the food delivery market share in Brazil, operating in more than 1500 cities and registering over 400,000 establishments on its platform [20]. Data from the same year indicate that the app facilitated 120 million orders per month [20]. In 2024, the company accounted for 0.55% of Brazil’s GDP, with more than 55 million consumers using the platform in the country [20]. Brazil’s prominence in this sector is noteworthy, with the highest revenue among Latin American countries and among the ten largest markets in the world [16].

Brazil is the largest country in South America, with an estimated population exceeding 210 million [21]. Belo Horizonte is the sixth most populous city in Brazil and the eighth in Latin America, with approximately 2.4 million inhabitants [21].

For this study, the addresses of the campuses of the two largest universities in Belo Horizonte were selected: the largest public institution (Federal University of Minas Gerais—UFMG) and the largest private institution (Pontifical Catholic University of Minas Gerais—PUC Minas). UFMG has 33,067 undergraduate students enrolled in on-site courses, 3167 faculty members, and 4694 administrative and technical staff distributed across its four campuses in Belo Horizonte (data provided by UFMG). PUC Minas has 26,514 undergraduate students, 1619 faculty members, and 1960 administrative staff members across its four campuses in the city [22].

The campuses were classified according to the number of undergraduate students as follows: main campus (public: 25,668; private: 14,022), secondary campus (public: 3797; private: 7036), tertiary campus (public: 2375; private: 3279), and quaternary campus (public: 1227; private: 2177) (data provided by UFMG) [22].

To compare app service at university and non-university addresses, one corresponding non-university location was selected for each university campus (yielding eight campus–location pairs). In this study, the term location refers to a single representative address point, which consists of the main entrance of the campus or a centrally selected address within the paired neighborhood. This comparison was undertaken to assess whether areas primarily frequented by young populations, such as university campuses, exhibit distinct patterns in meal-delivery app service. Previous studies indicate that younger age, student status, and higher demand for convenience, combined with limited available time, are associated with the use of these platforms [23,24].

The selection of corresponding non-university locations followed three criteria: location within the same administrative region, similar average income, and identical classification according to the Health Vulnerability Index (HVI). The HVI is an indicator used to guide and plan health actions, developed based on socioeconomic data from the Brazilian Institute of Geography and Statistics (IBGE) [25]. This summary indicator considers five components: sanitation conditions; housing; education; family income; health conditions; and age of the head of household. It categorizes neighborhoods in Belo Horizonte into four levels: low, medium, high, and very high [25]. Pairing with corresponding non-university locations aimed to control for potential regional differences, such as urban infrastructure and socioeconomic characteristics, which could influence accessibility to food delivery services.

**Figure 1:**
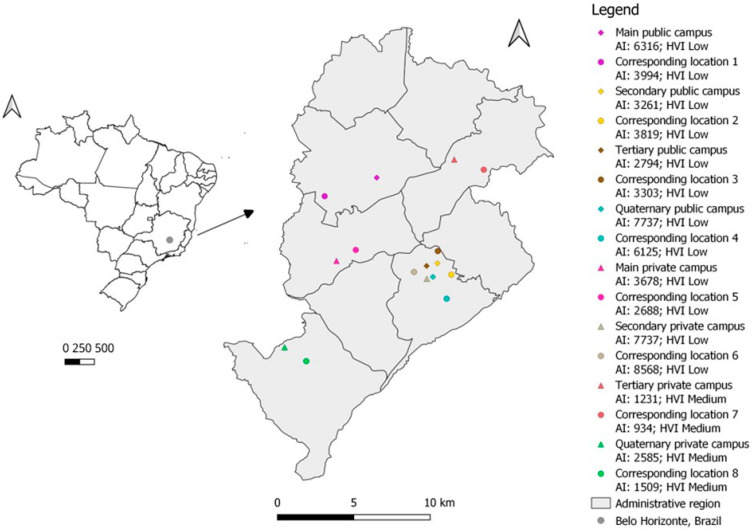
Spatial distribution and characteristics of the study sites, Belo Horizonte, Brazil, 2024. Note: Prepared by the authors. Symbols represent the study locations; color combinations indicate each university campus paired with its corresponding location (the central address of a neighborhood). Most campuses and paired sides are concentrated in the central region of the city. AI = average neighborhood income; HVI = Health Vulnerability Index.

The spatial distribution of public and private university campuses and their corresponding non-university locations, along with HVI and average income data, is presented in Figure 1. The map was created using QGIS software version 3.16.2. Eight addresses are located in the central region of Belo Horizonte, while the remainder are situated in non-central, peripheral areas.

### 2.2. Sample and Data Collection

The study sample comprised commercial establishments registered on the app that offered delivery services to the selected study locations. Data were obtained using an anonymous account to access the app’s website and simulate orders for each address. For university campuses, the main entrance address was used; for non-university locations, the central point of each neighborhood address as generated by the app was used.

Data collection took place on 29 and 30 April 2024 and was automated via web scraping. The variables collected included: establishment name, keyword used by the establishment, delivery distance between the establishment and the destination address (university or corresponding location), and delivery fee.

### 2.3. Organization of Variables

In total, 150,570 delivery options were identified across all study locations. As some establishments appeared multiple times for different addresses, a unique code was assigned to each address to distinguish delivery options.

Fifty-four distinct keywords were identified. After review, establishments associated with keywords such as beverages (n = 638), convenience (n = 441), gifts (n = 49), or market (n = 5) were excluded, as they did not sell ready-to-eat meals, the focus of this study. After exclusions, 149,437 delivery options remained, associated with 50 distinct keywords (distribution provided in Supplementary Material Table S1).

The keywords were grouped into eight categories based on similarity: Açaí and Ice Cream; Bakery Products and Desserts; International and Specialized Cuisine; Typical Brazilian Food; Meats, Fish, and Seafood; Snacks; Light Meals; and Others (keywords not fitting previous categories) (Table 1). This approach to classifying establishments based on keywords has been previously applied in a multi-city Latin American study [26] and in a comparative study in Chicago (USA), Melbourne (Australia), and Amsterdam (Netherlands) [8]. Keywords assist consumers in locating meals by type (e.g., dinner), cuisine (e.g., Brazilian), food item (e.g., pizza), or meal characteristics (e.g., healthy) [8].

Delivery distances were grouped into four categories: 0–1.6 km, greater than 1.6–4.8 km, greater than 4.8–8.0 km, and greater than 8.0 km. The 1.6 km (1 mile) threshold was chosen as it represents an approximate 20 min walking distance, according to the literature [27,28], with subsequent categories representing multiples of this value.

Delivery fees were categorized into BRL 0 (free delivery); greater than BRL 0–10 (USD greater than 0–1.90); greater than BRL 10–20 (USD greater than 1.90–3.81); and greater than BRL 20–30 (USD greater than 3.81–5.72). The April 2024 exchange rate (USD 1 = BRL 5.24) was used for conversion.

To determine the number of locations served by each establishment, the total appearances in the dataset were summed. Establishments with identical names but different keywords were treated as separate entities, as these variations represent distinct ways of presenting themselves to consumers within the app.

### 2.4. Data Analysis

Descriptive analyses were conducted to present the number of establishments and the distribution of keyword categories, delivery distances, and delivery fees for the total sample, as well as stratified by each university campus and its corresponding location. Absolute and relative frequencies were calculated, and 95% confidence intervals (CIs) were estimated for each variable. Differences between university and non-university locations were considered statistically significant when the 95% CIs did not overlap, corresponding to a significance level of 5%. Horizontal bar charts were used to illustrate these results. Complete results, including 95% CIs for each variable, are provided in Supplementary Materials Tables S2–S4. All analyses were performed using Stata software, version 14.0.

## 3. Results

The 149,437 establishments in the sample were repeatedly listed across the studied addresses, yielding a total of 19,315 unique establishments registered on the app. Of these, 40.6% (n = 7845) delivered to up to 4 different locations, whereas 28.7% (n = 5538) delivered to 13–16 locations (Table 2).

**Figure 2:**
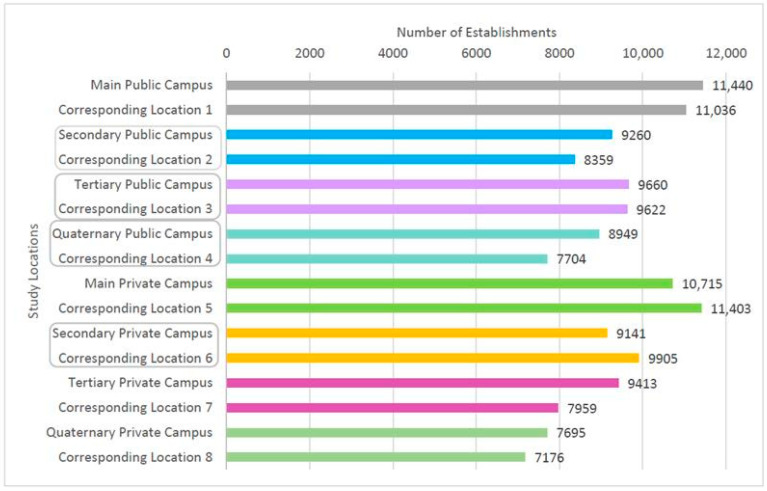
Number of food establishments available on the meal delivery app according to the study locations in Belo Horizonte, Brazil. Note: The study locations highlighted in the boxes are located in the central region of the city.

Excluding duplicates, the number of establishments delivering to each studied location ranged from 7176 (corresponding location 8) to 11,440 (main public campus). Delivery availability was generally higher at university campuses than at their corresponding locations, except for the main and secondary private campuses. Notably, the main public and private campuses, along with their corresponding peripheral locations (1 and 5), exhibited the greatest delivery coverage. Conversely, tertiary and quaternary private campuses and their corresponding locations (7 and 8), also in non-central areas, did not follow this pattern (Figure 2).

**Figure 3:**
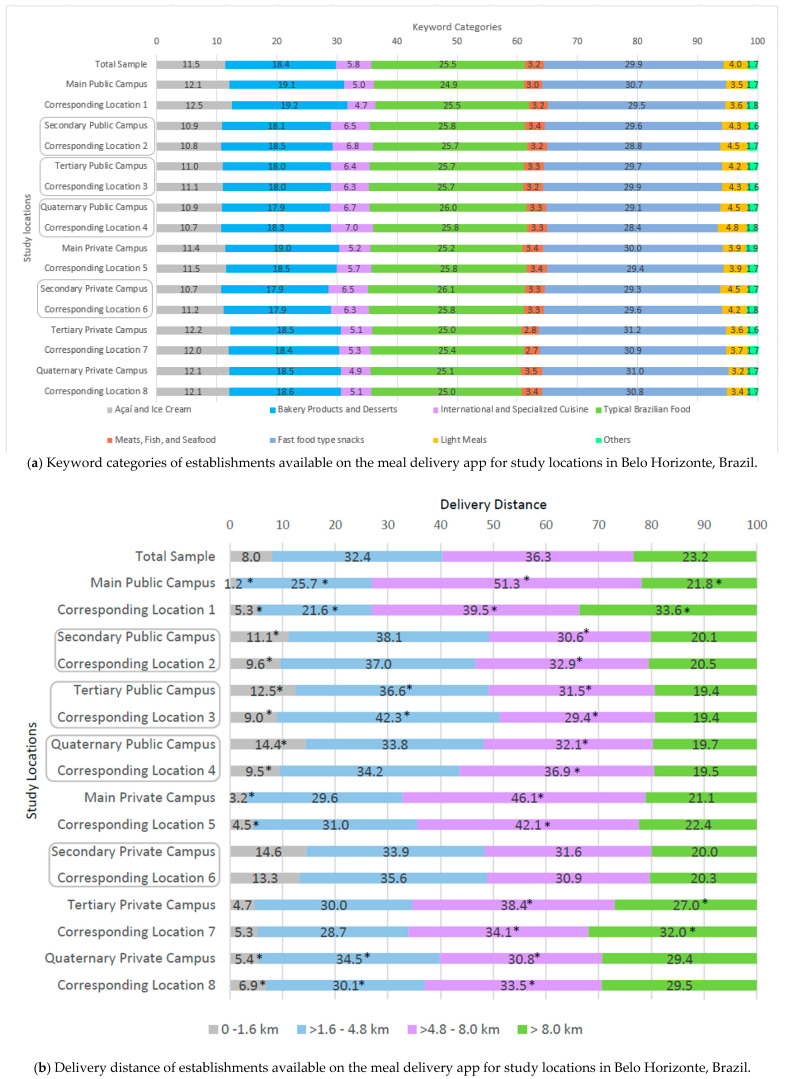

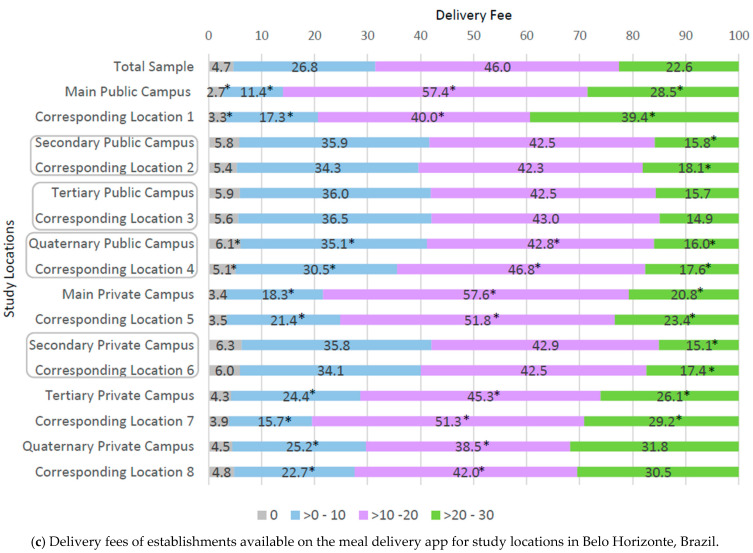
Distribution of establishments available on the meal delivery app and their characteristics according to the study locations in Belo Horizonte, Brazil. Note: The study locations highlighted in the boxes are located in the central region of the city. The asterisks (*) indicate values with significant differences according to the calculation of confidence intervals (CI 95%). In (**c**), consider the exchange rate of the dollar in April 2024 (USD 5.24) when making the conversion, as follows: BRL 0 (free delivery); BRL > 0–10 (USD > 0–1.90); BRL > 10–20 (USD > 1.90–3.81); BRL > 20–30 (USD > 3.81–5.72).

Among the keywords used by establishments, Snacks was the most frequent (29.9%), followed by Brazilian Typical Food (25.5%). In contrast, Light Meals (4.0%) and Meats, Fish, and Seafood (3.2%) were less common. This distribution was consistent across all study locations—both campuses and corresponding sites, in central and peripheral areas—indicating a relatively uniform keyword profile (Figure 3a; Supplementary Material Table S2).

Delivery distances ranged from 0 to 19.6 km. In the overall sample, 32.4% of establishments delivered within greater than 1.6–4.8 km, and 36.3% within greater than 4.8–8.0 km. Central locations, regardless of campus presence, showed a higher proportion of deliveries in the 0–1.6 km and greater than 1.6–4.8 km ranges, whereas peripheral areas had more deliveries in the greater than 4.8–8.0 km and greater than 8.0 km categories, particularly the main public and private campuses and their corresponding locations (1 and 5), as well as tertiary and quaternary private campuses and locations (7 and 8) (Figure 3b).

When comparing university campuses with their corresponding locations, most pairs showed a higher proportion of establishments delivering within the 0–1.6 km range at the campuses. Exceptions to this pattern included the main public and private campuses, as well as the quaternary private campus. In the 4.8–8.0 km range, delivery frequency was higher for the main public and private campuses and the tertiary public and private campuses compared to their corresponding locations. In the greater than 8 km category, two pairs (locations 1 and 7) received more deliveries than the main public and tertiary private campuses, respectively (Supplementary Material Table S3).

Delivery fees ranged from BRL 0 (free delivery) to BRL 30 (USD 5.72). Only a small proportion of establishments (4.7%) offered free delivery, while most (46.0%) charged BRL 10–20 (USD 1.90–3.81). Free delivery was more common at campuses and corresponding central locations compared to peripheral areas. Higher fees (greater than BRL 20–30; USD 3.81–5.72) predominated at campuses and locations further from the city center, irrespective of public or private status (Figure 3c).

When comparing campuses with corresponding locations, a higher proportion of establishments charging BRL 0–10 (USD 0–1.90) was observed for the quaternary public campus and tertiary and quaternary private campuses. Fees of BRL 10–20 (USD 1.90–3.81) were more frequent at corresponding locations 4, 7, and 8 than at the campuses. Similarly, the greater than BRL 20–30 (USD 3.81–5.72) category predominated at corresponding locations 1, 2, and 4–7 compared to the campuses (Supplementary Material Table S4).

## 4. Discussion

This study highlights the substantial number of establishments registered on a single online meal delivery platform serving both university and non-university environments within a major Brazilian city. Many establishments delivered to multiple addresses, reaching diverse areas. Across all locations, the keyword category Snacks predominated, with no significant differences observed in other categories. Delivery distances reached nearly 20 km, with the largest proportion serving peripheral areas, regardless of whether the locations were university-based. Free delivery was uncommon and more frequently concentrated in central areas, whereas higher delivery fees (greater than BRL 20/USD greater than 3.81) predominated in peripheral and non-university locations.

The university population, comprising students and workers, is influenced by the limited availability of food outlets on campus and in surrounding areas [11,29]. With technological advancements, these individuals have also gained the option to order meals via apps for on-campus consumption. Physical environmental factors, such as limited availability of healthy foods in workplaces, have been shown to determine higher app usage in such settings [29]. It is hypothesized that the same factors could apply to the university environment, where unhealthy food choices dominate due to limited physical and financial accessibility to healthy options [29,30,31,32,33], alongside a higher availability and promotion of ultra-processed foods [12,13,14,32]. The COVID-19 pandemic likely accelerated this trend. For example, a university in Rio de Janeiro experienced a decline in on-campus food establishments from 20 in 2019 to 14 in 2022, reducing the availability of fresh or minimally processed foods [14].

Our findings demonstrate that app-based services substantially expand the food environment in university settings, enabling the acquisition of meals and beverages without leaving campus. This convenience reduces time and effort while offering numerous options. To our knowledge, this is the first study to comprehensively characterize all establishments serving university and corresponding non-university environments, allowing for a detailed assessment of their reach. Previous studies have tended to focus only on popular establishments or those prioritized in search results presented to consumers [8,10,19,34].

We also show, for the first time in a middle-income country, that a single establishment can serve multiple addresses across different urban areas. While similar patterns have been documented in three high-income cities [8], our findings extend this phenomenon to new contexts. Notably, 92% of establishments delivered to locations beyond 1.6 km, consistent with distances reported in Canada (9.4 km) and China (10 km) [7,35]. It is important to note that some of these establishments may not have a physical location, such as dark kitchens, which operate exclusively via apps and lack a public-facing space [36], further expanding the digital food environment.

In practice, the app eliminates the need for customers to visit physical outlets, delivering food directly to their location. Central areas and campuses received more short-distance deliveries, likely due to higher commercial density and the presence of physical establishments that also use aggregator platforms.

Despite the large number of establishments, keyword diversity was limited. Snacks dominated regardless of geographic location or university status. This pattern is consistent with observations from high-income countries [8] and Latin America [26], and with studies showing that unhealthy options predominate in app-based menus in Brazil [10,26] and abroad [4,7,19,34,37,38].

Such uniformity suggests that delivery apps homogenize the food environment, in contrast to the physical environment, where food access often varies according to socioeconomic status and geography. For instance, in Baltimore, USA, fewer healthy food outlets were found within 1.6 km of low-income neighborhoods, potentially affecting diet quality [27].

Regarding delivery fees, free delivery was rare. Higher proportions were reported in São Paulo on another platform [26] and in other Latin American cities [26], suggesting that promotional strategies vary by platform and location. Higher fees were more frequent in peripheral areas, likely reflecting longer travel distances. University campuses generally had lower fees, possibly as a targeted pricing strategy, given that cost is a key determinant of service use and food choice among students and young adults [17,19,24,39].

These results support previous findings that the digital food environment influences access to food [8,35] and highlight the high exposure of university populations to app-based services. The combination of abundant unhealthy options and elevated delivery costs may limit healthy eating through apps, echoing patterns found in the physical environment [31]. Future research should integrate analyses of both environments, as delivery services may contribute to obesogenic contexts [35].

Importantly, such environments are not static: they can be managed and regulated. For example, universities could strengthen their food environment by promoting affordable and healthy on-campus alternatives, such as university restaurants that prioritize unprocessed or minimally processed foods, while also discouraging excessive reliance on delivery apps. Educational campaigns could complement these measures by supporting healthier choices among app users. At the same time, the implications of meal delivery apps extend beyond nutrition. Long delivery distances, such as those observed in this study, may contribute to environmental impacts through increased fuel consumption. Moreover, these platforms may discourage walking to restaurants or preparing meals at home, reinforcing sedentary lifestyles among university populations who already spend long hours seated for studying, attending classes, or working. The expansive nature of delivery apps also challenges the traditional concept of “local food environments,” typically defined as outlets located within a 1 km radius of one’s residence. Individuals are now experiencing “hybrid food environments” that combine physical and digital access, which may exacerbate exposure to unhealthy foods [40].

Currently, many meal delivery platforms operate with little or no regulation, despite public health calls for stronger governmental action in this sector. Several strategies could be applied within digital platforms, including the implementation of menu labeling for online menus, restrictions on the marketing of unhealthy foods, financial incentives to promote healthier options, and the use of algorithms that prioritize healthier choices [2,40]. Ultimately, policies that integrate digital food environments into the broader food policy agenda are essential to mitigate risks and to ensure that these platforms support, rather than undermine, global nutrition and chronic disease prevention goals [40].

Strengths of this study include the inclusion of all establishments across study locations, without restrictions by day or meal time, and the direct comparison between university and non-university environments. Limitations include reliance on anonymous browsing for delivery fee data, which does not account for personalized discounts, and the use of establishment keywords instead of direct menu analysis, which may not accurately reflect available items. Although this study paired areas with and without universities while controlling for administrative region, income, and the HVI, we acknowledge that additional unmeasured characteristics may persist and potentially influence the findings. Further studies should explore user perceptions of app-based offerings, particularly in university settings.

## 5. Conclusions

Food delivery apps are widely accessible in both university and non-university environments, characterized by high establishment availability and extensive delivery reach. By extending service far beyond neighborhood boundaries, these platforms penetrate peripheral areas, although with higher delivery costs. The predominance of unhealthy food categories such as Snacks was consistent across locations. Given the popularity and convenience of these services, university administrators should implement policies to promote healthy food environments by increasing the availability of affordable, nutritious options, thereby reducing reliance on delivery apps.

## Figures and Tables

**Table 1:** Categorization of keywords used by establishments on the meal delivery app (n = 50).

Categories	Keywords
Açaí and Ice Cream	Açaí—Ice Cream
Bakery Products and Desserts	Cafeteria—Desserts and Cakes—Bakery
International and Specialized Cuisine	African—German—Arab—Argentine—Asian—Chinese—Colombian—Contemporary—Spanish—French—Indian—Italian—Japanese—Mediterranean—Mexican—Peruvian—Portuguese
Typical Brazilian Food	Baiana—Brazilian—Gaúcha—Marmita—Mineira—Northeastern
Meats, Fish, and Seafood	Meats—Chickens—Seafood—Fish
Snacks	Burger—Snacks—Savory Snacks—Pizza—Savory Pastry—Xis
Light Meals	Crepes—Healthy Frozen Meals—Juice Bar—Pancakes—Healthy—Soups and Broths—Tapioca—Vegan—Vegetarian
Others	Frozen Foods—Fast Cuisine—Varied

**Table 2:** Coverage of food establishments according to delivery addresses in Belo Horizonte, Brazil.

Number of Addresses	Number of Establishments	%
1–4	7845	40.6
5–8	2822	14.6
9–12	3110	16.1
13–16	5538	28.7
Total	19,315	100

## Data Availability

Data will be made available on request.

## Supplementary Materials

The following supporting information can be downloaded at: https://www.mdpi.com/article/10.3390/ijerph22091448/s1, Table S1: Distribution of keywords used by establishments available on the meal delivery app in Belo Horizonte, Brazil; Table S2: Distribution of establishments available on the meal delivery app according to keywords and study locations in Belo Horizonte, Brazil; Table S3: Distribution of establishments available on the meal delivery app according to distance categories and study locations in Belo Horizonte, Brazil; Table S4: Distribution of establishments available on the meal delivery app according to delivery fee categories and study locations in Belo Horizonte, Brazil.

## Abbreviations

The following abbreviations are used in this manuscript:

Apps	Meal delivery apps
HVI	Health Vulnerability Index
