# Supplementary material for: Access to Food Establishments via Meal Delivery Applications: A Study of University and Non-University Settings in a Brazilian Metropolis
Source: Int J Environ Res Public Health. 2025 Sep 18;22(9):1448. doi: 10.3390/ijerph22091448 (PMC12469746; doi:10.3390/ijerph22091448)
Supplement: Supplementary file 1 [file ijerph-22-01448-s001.zip › ijerph-3847383-supplementary.pdf]

Supplementary Material Table S1: Distribution of keywords used by establishments available on the meal delivery app in Belo Horizonte, Brazil

Categories	Keywords	n	%
**Açaí and Ice Cream**	Açaí	13,877	9.29
	Ice Cream	3,235	2.16
**Bakery Products and Desserts**	Café	1,865	1.25
	Desserts & Cakes	22,516	15.07
	Bakery	3,128	2.09
**International and Specialized Cuisine**	African	38	0.03
	German	46	0.03
	Arab	673	0.45
	Argentine	120	0.08
	Asian	8	0.01
	Chinese	397	0.27
	Colombian	27	0.02
	Contemporary	225	0.15
	Spanish	22	0.01
	French	175	0.12
	Indian	127	0.08
	Italian	2,762	1.85
	Japanese	3,595	2.41
	Mediterranean	91	0.06
	Mexican	285	0.19
	Peruvian	33	0.02
	Portuguese	62	0.04
**Typical Brazilian Food**	Baiana	27	0.02
	Brazilian	27,785	18.59
	Gaúcha	10	0.01
	Meal Box	9,305	6.23
	Mineira	942	0.63
	Northeastern	78	0.05
**Meats, Fish, and Seafood**	Meats	3,74	2.50
	Chickens	298	0.20
	Seafood	370	0.25
	Fish	432	0.29
**Snacks**	Burger	3,809	2.55
	Snacks	27,769	18.58
	Savory pastry	1,372	0.92
	Pizza	7,961	5.33
	Savory Snacks	3,702	2.48
	Xis	14	0.01
**Light Meals**	Crepe	126	0.08
	Healthy frozen foods	54	0.04
	Juice Bar	196	0.13
	Pancake	3	0.00
	Healthy	4,430	2.96

	Soups & Broths	266	0.18
	Tapioca	15	0.01
	Vegan	447	0.30
	Vegetarian	424	0.28
**Others**	Frozen Foods	1,01	0.68
	Fast Cuisine	623	0.42
	Varied	922	0.62
**Total**	-	149,437	100

Supplementary Material Table S2: Distribution of establishments available on the meal delivery app according to keywords and study locations in Belo Horizonte, Brazil

Locations	Açaí and Ice Cream		Bakery Products and Desserts		International and Specialized Cuisine		Typical Brazilian Food		Meats, Fish, and Seafood		Snacks		Light Meals		Others	
	**%**	**95% CI**	**%**	**95% CI**	**%**	**95% CI**	**%**	**95% CI**	**%**	**95% CI**	**%**	**95% CI**	**%**	**95% CI**	**%**	**95% CI**
Main Public Campus	12.08	11.49 - 12.69	19.09	18.38 - 19.82	4.97	4.59 -5.38	24.94	24.15 - 25.74	3.00	2.70 - 3.32	30.72	29.87 - 31.56	3.50	3.17 - 3.84	1.70	1.48 - 1.95
Corresponding Location 1	12.49	11.88 - 13.11	19.19	18.46 - 19.93	4.72	4.34 - 5.13	25.51	24.70 - 26.32	3.20	2.88 -3.54	29.54	28.69 - 30.39	3.60	3.26 - 3.96	1.76	1.52 -2.02
**Secondary Public Campus**	10.91	10.28 - 11.55	18.05	17.27 - 18.84	6.46	5.97 -6.97	25.81	24.92 - 26.71	3.35	3.00 - 3.73	29.55	28.62 - 30.48	4.27	3.87 - 4.69	1.62	1.38 - 1.89
**Corresponding Location 2**	10.77	10.12 - 11.44	18.48	17.66 - 19.32	6.76	6.24 - 7.31	25.67	24.74 - 26.62	3.23	2.87 - 3.63	28.82	27.85 - 29.80	4.53	4.10 -5.00	1.73	1.47 - 2.03
**Tertiary Public Campus**	10.97	10.36 - 11.61	18.01	17.25 - 18.79	6.42	5.94 - 6.92	25.72	24.86 - 26.60	3.32	2.98 - 3.69	29.66	28.75 - 30.57	4.22	3.84 -4.64	1.67	1.42 - 1.94
**Corresponding Location 3**	11.05	10.43 - 11.68	17.99	17.23 - 18.77	6.26	5.78 - 6.75	25.68	24.81 - 26.56	3.24	2.90 - 3.61	29.91	29.00 - 30.83	4.26	3.87 - 4.68	1.61	1.37 - 1.88
**Quaternary Public Campus**	10.91	10.27 - 11.56	17.91	17.13 - 18.72	6.69	6.19 – 7.23	26.00	25.10 - 26.92	3.27	2.92 – 3.66	29.06	28.13 - 30.01	4.49	4.08 - 4.94	1.65	1.40 - 1.93
**Corresponding Location 4**	10.68	10.01 - 11.39	18.28	17.42 - 19.15	7.00	6.44 -7.58	25.77	24.80 - 26.75	3.26	2.88 -3.67	28.43	27.43 - 29.44	4.75	4.29 -5.24	1.84	1.56 -2.16
Main Private Campus	11.43	10.84 12.04	18.95	18.22 - 19.70	5.22	4.81 - 5.65	25.19	24.37 26.01	3.44	3.11 - 3.80	30.00	29.14 - 30.87	3.88	3.53 - 4.26	1.88	1.63 - 2.15
Corresponding Location 5	11.51	10.93 - 12.10	18.51	17.81 - 19.23	5.67	5.25 - 6.10	25.83	25.03 - 26.63	3.43	3.11 -3.77	29.41	28.58 - 30.25	3.91	3.57 - 4.28	1.74	1.51 -1.99
**Secondary Private Campus**	10.70	10.08 - 11.34	17.90	17.12 - 18.69	6.53	6.04 - 7.05	26.08	25.19 - 26.99	3.30	2.95 - 3.69	29.31	28.38 - 30.24	4.45	4.04 - 4.89	1.73	1.48 - 2.01
**Corresponding Location 6**	11.16	10.55 - 11.79	17.87	17.12 - 18.63	6.32	5.85 – 6.81	25.76	24.91 - 26.63	3.32	2.98 - 3.69	29.63	28.74 - 30.53	4.15	3.77 - 4.56	1.79	1.54 - 2.06
**Tertiary Private Campus**	12.20	11.54 - 12.87	18.47	17.70 - 19.27	5.13	4.70 - 5.59	24.97	24.10 - 25.85	2.78	2.46 - 3.13	31.20	30.27 - 32.14	3.63	3.27 - 4.03	1.61	1.37 - 1.89
**Corresponding Location 7**	11.96	11.26 - 12.69	18.36	17.52 - 19.22	5.30	4.83 - 5.81	25.44	24.49 - 26.41	2.73	2.39 - 3.10	30.90	29.88 - 31.92	3.66	3.26 - 4.09	1.66	1.40 - 1.96
Quaternary Private Campus	12.11	11.40 – 12.86	18.54	17.69 - 19.42	4.87	4.41 - 5.37	25.06	24.09 - 26.03	3.51	3.11 - 3.94	31.03	30.00 - 32.07	3.22	2.85 - 3.64	1.65	1.38 - 1.96
Corresponding Location 8	12.10	11.36 - 12.87	18.59	17.70 - 19.50	5.09	4.60 - 5.61	24.97	23.98 - 25.98	3.44	3.04 - 3.88	30.76	29.69 - 31.83	3.39	2.99 - 3.83	1.67	1.39 - 1.99

Note: The study locations highlighted in bold represent the locations situated in the central region of the city.

Supplementary Material Table S3: Distribution of establishments available on the meal delivery app according to distance categories and study locations in Belo Horizonte, Brazil.

Locations	Delivery Distance (km)							
	0 – 1.6		1.6 – 4.8		4.8 – 8.0		> 8.0	
	%	95% CI	%	95% CI	%	95% CI	%	95% CI
Main Public Campus	1.21*	1.02 - 1.42	25.70*	24.90 - 26.50	51.26*	50.34 - 52.17	21.84*	21.08 - 22.60
Corresponding Location 1	5.32*	4.91 - 5.75	21.60*	20.84 - 22.37	39.45*	38.54 - 40.36	33.63*	32.75 - 34.51
**Secondary Public Campus**	11.14*	10.51 - 11.80	38.11	37.12 - 39.10	30.64*	29.70 - 31.58	20.11	19.30 - 20.93
**Corresponding Location 2**	9.64*	9.02 - 10.29	37.01	35.98 - 38.05	32.90*	31.89 - 33.91	20.45	19.59 - 21.32
**Tertiary Public Campus**	12.52*	11.87 - 13.19	36.61*	35.65 - 37.58	31.50*	30.58 - 32.43	19.37	18.59 - 20.16
**Corresponding Location 3**	8.98*	8.42 - 9.56	42.28*	41.29 - 43.26	29.38*	28.47 - 30.29	19.36	18.58 - 20.16
**Quaternary Public Campus**	14.40*	13.69 - 15.14	33.78	32.80 - 34.76	32.13*	31.16 - 33.10	19.69	18.87 - 20.52
**Corresponding Location 4**	9.48*	8.84 - 10.15	34.19	33.13 - 35.25	36.86*	35.79 - 37.94	19.47	18.60 - 20.37
Main Private Campus	3.19*	2.87 - 3.54	29.60	28.74 - 30.47	46.13*	45.18 - 47.07	21.07	20.31 - 21.85
Corresponding Location 5	4.52*	4.15 - 4.91	31.03	30.18 - 31.88	42.10*	41.19 - 43.01	22.35	21.59 - 23.12
**Secondary Private Campus**	14.56	13.85 - 15.29	33.85	32.88 - 34.82	31.64	30.69 - 32.59	19.95	19.14 - 20.78
**Corresponding Location 6**	13.25	12.59 - 13.92	35.57	34.63 - 36.51	30.88	29.98 - 31.80	20.30	19.52 -21.10
**Tertiary Private Campus**	4.70	4.28 - 5.14	29.95	29.03 - 30.88	38.40*	37.42 - 39.39	26.95*	26.06 - 27.85
**Corresponding Location 7**	5.28	4.80 - 5.79	28.65	27.66 - 29.65	34.11*	33.07 - 35.16	31.96*	30.94 - 32.99
Quaternary Private Campus	5.41*	4.92 - 5.93	34.46*	33.40 - 35.53	30.77*	29.75 - 31.81	29.36	28.34 - 30.38
Corresponding Location 8	6.87*	6.30 - 7.47	30.13*	29.07 - 31.20	33.54*	32.45 - 34.64	29.46	28.41 - 30.52

Note: The study locations highlighted in bold represent the locations situated in the central region of the city. The asterisks (*) indicate values with significant differences according to the calculation of confidence intervals (CI 95%).

Supplementary Material Table S4: Distribution of establishments available on the meal delivery app according to delivery fee categories and study locations in Belo Horizonte, Brazil.

**Locations**	**Delivery Fees (R$)**							
	**0**		**>0-10**		**>10-20**		**>20-30**	
	**%**	**95% CI**	**%**	**95% CI**	**%**	**95% CI**	**%**	**95% CI**
Main Public Campus	2.68*	2.40 - 2.99	11.34*	10.76 - 11.93	57.57 *	56.66 - 58.47	28.41*	27.58 - 29.24
Corresponding Location 1	3.33*	3.01 - 3.68	17.31*	16.61 - 18.02	40.00*	39.08 - 40.91	39.36*	38.45 - 40.27
**Secondary Public Campus**	5.78	5.32 - 6.27	35.89	34.91 - 36.86	42.52	41.51 - 43.52	15.82*	15.09 - 16.57
**Corresponding Location 2**	5.36	4.89 - 5.86	34.25	33.24 - 35.27	42.28	41.22 - 43.34	18.11*	17.30 - 18.95
**Tertiary Public Campus**	5.86	5.40 - 6.34	36.04	35.08 - 36.99	42.45	41.47 - 43.44	15.65	14.94 - 16.39
**Corresponding Location 3**	5.61	5.16 - 6.09	36.46	35.50 - 37.42	43.02	42.02 - 44.00	14.91	14.21 - 15.63
**Quaternary Public Campus**	6.09*	5.61 - 6.60	35.09*	34.10 - 36.08	42.84*	41.82 - 43.87	15.98*	15.23 - 16.75
**Corresponding Location 4**	5.05*	4.58 - 5.56	30.53*	29.51 - 31.56	46.78*	45.66 - 47.89	17.64*	16.80 - 18.50
Main Private Campus	3.37	3.04 - 3.72	18.25*	17.53 - 18.99	57.62*	56.68 - 58.55	20.76*	19.99 - 21.53
**Corresponding Location 5**	3.46	3.14 - 3.81	21.37*	20.62 - 22.13	51.77*	50.84 - 52.68	23.40*	22.62 - 24.18
**Secondary Private Campus**	6.25	5.76 - 6.76	35.84	34.86 - 36.82	42.85	41.83 - 43.86	15.06*	14.34 - 15.81
**Corresponding Location 6**	5.95	5.49 - 6.42	34.11	33.18 - 35.05	42.53	41.56 - 43.51	17.41*	16.67 - 18.16
**Tertiary Private Campus**	4.25	3.86 - 4.67	24.39*	23.53 - 25.26	45.26*	44.25 - 46.26	26.10*	25.22 - 26.99
**Corresponding Location 7**	3.87	3.46 - 4.31	15.67*	14.88 - 16.48	51.30*	50.20 - 52.39	29.16*	28.17 - 30.17
**Quaternary Private Campus**	4.48	4.04 - 4.96	25.20*	24.24 - 26.18	38.53*	37.44 - 39.62	31.79	30.75 - 32.83
**Corresponding Location 8**	4.84	4.36 - 5.35	22.73*	21.77 - 23.71	41.97 *	40.83 - 43.11	30.46	29.40 - 31.53

Note: The study locations highlighted in bold represent the locations situated in the central region of the city. The asterisks (*) indicate values with significant differences according to the calculation of confidence intervals (CI 95%).
